# Ambulatory 24-hour multichannel intraluminal impedance-pH monitoring and high resolution endoscopy distinguish patients with non-erosive reflux disease from those with functional heartburn

**DOI:** 10.1371/journal.pone.0175263

**Published:** 2017-04-06

**Authors:** Chuanlian Chu, Quanlin Du, Changqing Li, Linlu Zhang, Xiaoyan Zhou, Fang Zuo, Yanmin Zhang, Fang Li, Guofeng Xie, Yanqing Li

**Affiliations:** 1 Department of Gastroenterology, Jinan Central Hospital, Shandong University, Jinan, Shandong province, China; 2 Department of Gastroenterology, Qilu Hospital, Shandong University, Jinan, Shandong province, China; 3 Division of Gastroenterology and Hepatology, University of Maryland School of Medicine, Maryland VA Health Care System, Baltimore, Maryland, United States of America; University Hospital Llandough, UNITED KINGDOM

## Abstract

**Aims:**

To assess the contribution of 24-h esophageal multichannel intraluminal impedance and pH (MII-pH) monitoring and high resolution endoscopy (HRE) with i-scan imaging in differentiating non erosive reflux disease (NERD) from functional heartburn (FH).

**Methods:**

This is a retrospective cohort study of patients with heartburn from the Endoscopy Unit. NERD patients and FH patients were defined by 24-h MII-pH monitoring and white light endoscopy. Minimal mucosal changes were assessed by HRE with i-scan imaging.

**Results:**

Total of 156 consecutive patients with heartburn but without esophageal mucosal erosions were studied. Forty-eight of these subjects had NERD, with increased acid exposure time (AET) and positive SAP and/or SI. Eighteen had FH with normal AET and negative SAP and SI. When compared to FH patients and healthy controls, NERD patients had significantly increased number of total acid reflux episodes, as well as increased number of weakly acidic reflux episodes (*p*<0.01). The rate of proximal reflux episodes in NERD patients was higher than that of FH patients and healthy controls (*p*<0.01). Irregular or blurring of the Z-line (58.3%) and white mucosal turbidity (47.9%) were the most common endoscopic findings of minimal mucosal changes observed in this study. NERD patients had more prevalent minimal changes than FH patients and the controls (87.5%*vs*. 66.6%vs. 61.9%; *p* = 0.004) with sensitivity of 87.5%. Histopathological evaluation showed that NERD patients had significantly higher average scores of intercellular spaces dilation (2.82±0.9 vs. 1.2±0.6, p = 0.005) and papillae elongation (2.65±1.0 vs. 1.5±0.8, *p* = 0.014), but not for basal cell proliferation (1.6±1.3 vs. 1.0±0.9, *p* = 0.070). The histological scores of the NERD patients were 7.1±1.2, which were higher than those of FH patients (3.4±1.0, *p* = 0.004).

**Conclusions:**

Minimal mucosal changes could be useful markers to support clinical diagnosis of NERD. Combination of 24-h MII-pH monitoring and i-scan high resolution endoscopy can distinguish patients with NERD from those with FH.

## Introduction

The symptom of heartburn, defined as “a burning sensation behind the breastbone”, is associated with the occurrence of acid reflux events in most patients [1, 2]. Traditionally, heartburn is considered as the cardinal manifestation of gastro-esophageal reflux disease (GERD), which allows clinical diagnosis without the need for any further invasive investigations [3]. However, patients with functional heartburn (FH), another common esophageal disease, also have symptom of heartburn, but without proven gastro-esophageal reflux [4, 5]. Patients with these two disorders share similar clinical manifestations, including normal esophageal appearance on traditional endoscopy and unsatisfactory response to acid suppressive therapy [4], particularly in cases with absent histopathology-based esophageal motility disorders. Unfortunately, treatment of FH remains an individual approach in most clinical practice and largely empirical due to its poor response to acid suppressive therapy and accompanying psychopathological component [6]. Therefore, monitoring heartburn in patients primarily diagnosed with NERD allows one to distinguish them from those with true FH.

Multichannel intraluminal impedance and pH (MII-pH) monitoring is a novel technique that allows characterization of acid exposure and assessment of extent of reflux in the proximal esophagus. Combined with symptom association probability (SAP), MII-pH monitoring permits one to characterize heartburn with more accuracy and to also explore the role of weakly acidic reflux events in provoking symptoms in patients with NERD and FH [7, 8]. This technique allows stratification of heterogeneous symptoms from NERD or FH patients with heartburn but negative endoscopic findings and separation of these patients into NERD or functional heartburn groups.

Although it is debatable [9], more and more gastroenterologists accept minimal changes as positive endoscopic findings for patients with NERD [10–13]. Minimal mucosal changes such as erythema, edema, irregular or blurring of the Z-line, friability, and white mucosal turbidity have been evaluated in patients with NERD [9, 14], functional dyspepsia [15, 16] and in healthy subjects [10]. Lei et al. [14] showed that the presence of minimal change esophagitis was associated with NERD and indicated esophageal acid exposure. To distinguish NERD from FH patients, Luo et al. [17] used autofluorescence imaging (AFI) endoscopy to observe inconspicuous lesions. They showed that the presence of purple lines in the distal esophagus on AFI were observed in 90.5%of patients with NERD, but only in 10% of FH patient. The sensitivity and specificity of AFI in differentiating NERD from FH were 90.5% and 90.0%, respectively.

I-scan endoscopy is a new digital chromo-endoscopy optical enhancement technique. It is software-based real-time modification of image sharpness, hue and contrast provides high resolution images that improve identification of minimal change lesions [18]. Kim et al. [19] showed that i-scan endoscopy identified more minimal changes than conventional endoscopy and therefore increased the efficiency of GERD diagnosis. More recently, a cohort study was conducted to assess the efficacy of i-scan endoscopy in detecting minimal change lesions in dyspeptic patients with or without GERD [20]. They showed that i-scan endoscopy detected more minimal change esophagitis in GERD patients than non-GERD patients, albeit with a low sensitivity and specificity. Hence, whether i-scan endoscopy is capable of differentiating NERD from FH remain to be determined.

In the present study, in a series of patients with heartburn but negative conventional endoscopic findings, we retrospectively compared the characteristics of reflux episodes using MII-pH monitoring and high resolution endoscopy (HRE) with i-scan to test the efficacy of i-scan endoscopy for detecting minimal changes. We also want to determine whether observed differences using MII-pH monitoring and high resolution endoscopy allow us to distinguish NERD from FH.

## Materials and methods

### Study design

We retrospectively studied a series of patients from a prospectively-established database with typical GERD symptoms, i.e., heartburn lasting for more than 6 months and occurring at least three times weekly, from the endoscopy unit of Qilu Hospital between April 2010 and December 2014. Consecutive outpatients with GERD symptoms were recruited.

Based on the results obtained from 24-h MII-pH monitoring and white light endoscopy, the subjects were classified into two groups according to the Rome III criteria [4]: (1) NERD group were defined with typical reflux symptoms, negative upper endoscopy and abnormal esophageal acid exposure from impedance–pH monitoring. (2) Diagnosis of FH was made if there was normal endoscopic appearance of the gastro-esophageal junction in combination with a normal acid exposure time (AET) without any symptom association (negative SI and SAP). Hypersensitive esophagus was defined as having normal AET and number of reflux episodes but positive SI or SAP. Patients without any reflux symptoms with negative endoscopic findings were recruited as healthy controls.

Exclusion criteria were as follows: age <18 years, reflux esophagitis, Barrett’s esophagus, esophageal varices, evidence of cancer or mass lesion in the esophagus, gastric lesions (ulcer, cardiac polyp, cancer), previous thoracic, esophageal, or gastric surgery, significant untreated medical conditions, history of alcohol or drug abuse, or severe uncontrolled coagulopathy. The study protocol was approved by the Medical Ethics Committee of Qilu Hospital, Shandong University.

### Technique of 24h MII-pH monitoring

All 24-h MII-pH monitoring procedures performed in patients and controls used a 2.1-mmdiameter catheter (Medical Measurement Systems B.V., Enschede, Holland). The catheter positions in the distal esophagus were determined using the pH step-up method [21]. The lower four impedance sensors were positioned at the distal esophagus (3, 5, 7 and 9 cm above the LES), and the upper two measuring sensors placed at the proximal esophagus (15 and 17 cm above the LES) respectively to measure the impedance data. All patients and subjects were provided with and instructed to keep a diary to record exact timing of meals, supine and upright positions, as well as symptoms such as heartburn, regurgitation and non-cardiac chest pain. Patients had no restrictions on meals except for food with pH<4. Data from MII-pH monitoring were uploaded and analyzed using dedicated software (BioView Analysis; Sandhill Scientific, USA). All data were analyzed by one investigator who was blinded to patients’ clinical information.

### Gastro-esophageal reflux parameters

The variables of the impedance signals included total number of reflux episodes in terms of composition (liquid, gas, and mixed reflux episodes), and pH (acidic, weakly acidic, and non-acidic), total number of reflux episodes, AET (% time with esophageal pH<4), proximal extent of the refluxate, symptom association probability (SAP), symptom index (SI) and symptomatic response to proton pump inhibitors (PPI). The criteria for these parameters were described previously by others [22–24]. SAP values of ≥ 95% are considered positive [25].

### I-scan endoscopy procedure

All patients underwent high resolution white light endoscopy using i-scan technology (Pentax, EC, -3890i, Tokyo, Japan). Preparation of patients for endoscopic procedure was similar to that of standard upper endoscopy. During the procedures, the upper gastrointestinal tract was first carefully visualized using standard white light endoscopy alone without i-scan. Subsequently, the tone enhancement esophageal mode was activated by pressing the corresponding button at the endoscope control head and the lower esophagus and the gastro-esophageal junction was re-inspected. The shape of Z-line and mucosal appearance near gastro-esophageal junction was observed. When analyses was performed, the stored esophageal images were carefully evaluated based on established endoscopic criteria: blurring or irregular Z-line, erythema, edema, friability, white turbid discoloration, and/or accentuation of mucosal folds according to the LA classification system with Japanese modifications and the other findings of minimal changes [9, 26–28]. At the end of the procedures, biopsy specimens were obtained from lower esophagus of each individual and were sent for histopathologic examination.

### Histopathologic evaluation

For histopathology, we retrospectively examined microscopic changes of the distal esophageal epithelium including dilated intercellular spaces, elongation of the papillae and proliferation of basal cells according to the histological severity scores established by Kandulski et al. in 2013 [29]. The degrees of these microscopic changes were re-assessed and semi-quantitatively scored as 0 (absent), 1 (mild), 2 (moderate), or 3 (severe) on hematoxylin–eosin-stained slides. Histopathologic evaluation was performed by one investigator who was blinded to patient information.

### Statistical analysis

Data were expressed as mean±SD. Comparisons between continuous variables were analyzed using paired Student’s *t*-test at each esophageal level. Differences in proportions and categorical variables were compared using the chi-square or Fisher’s exact test. Statistical analyses were performed using SPSS 16.0 for Windows (SPSS Inc., Chicago, IL). Differences were considered statistically significant when *p*<0.05. Multivariate regression analysis was performed to analyze the relationships among impedance-pH features, HRE and histologic changes.

## Results

### Patient characteristics

**Fig 1:**
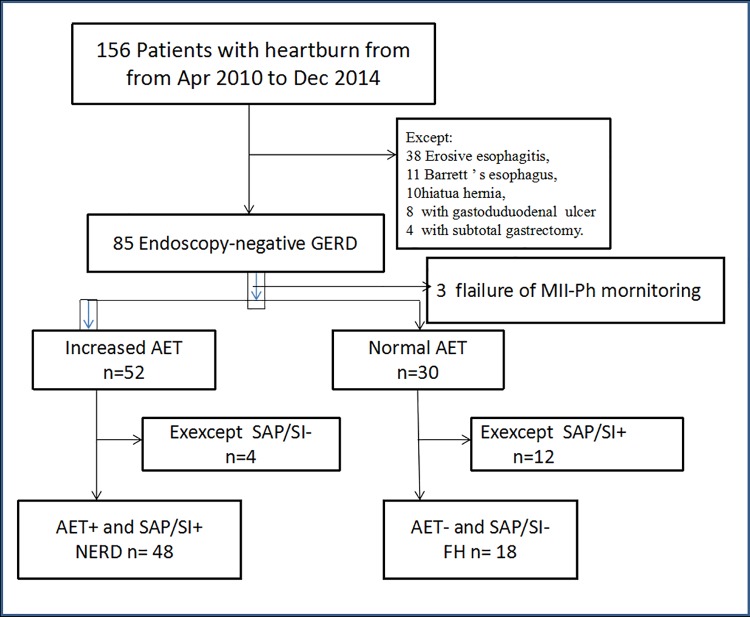
Study design and the disposition of patients.

As shown in Fig 1, overall, 103 participants, including 82 subjects with heartburn and 21 healthy controls, were eligible for final analysis. According to combined MII-pH monitoring, 48 (58.5%) had positive SAP and/or positive SI (NERD group). Thirty patients had normal distal esophageal AETs, and 18(21.9%) of those had negative SAP and SI (FH group). Twelve patients (14.6%) from this group had hyper sensitive esophagus (normal AET and positive SAP and/or SI) and were excluded (Fig 1).

As shown in Table 1, the demographic data showed that there was no difference in the mean ages of patients with NERD (22 men, mean age 49.4 years; range 34–59) and FH (8 men, mean age 46.3 years; range 33–61) with those of subjects in the control group (*p*>0.05). The mean body mass index (BMI) was not higher in the NERD group and FH group compared with controls (*p* = 0.536). The DeMeester scores were higher for NERD patients than FH patients and controls (48±37 *vs*. 24±19 and 20±17, *p* = 0.002). The AET of NERD group (7.3±2.9) was higher than that of FH (4.3±2.7) and controls (3.9±2.8) (*p* = 0.001). Similar results of SAP (66.7% *vs*. 22.2% and 16.7%, *p* = 0.001) and positive SI (52.1% vs.22.2% and 9.5%, *p* = 0.001) were found between the two groups. As for the reflux episodes, NERD patients had a significantly increased number of total and acid reflux episodes (*p*<0.01) when compared with FH, but not for the number of weakly acidic reflux and gas episodes (*p*>0.05). The rate of proximal reflux episodes in patients with NERD was higher than that of FH patients and the controls (52.6% vs. 22.3% and 16.7%, *p*< 0.01; Table 1).

**Table 1:** Demographics and clinical characteristics of patients with non-erosive reflux disease (NERD) were classified based on acid exposure time (AET) and symptom association probability (SAP).

Demographics and clinical parameters	NERD (n = 48)	FH (n = 18)	Controls (n = 21)	*p* value
**Mean age (yrs, mean±SD)**	49.4±6.9	45.1±5.2	40.6±2.3	0.923
**Males, n (%)**	22 (45.8)	8 (44.4)	11 (52.4)	0.799
**Mean body mass index (BMI)**	22.9±5.6	20.2±44	19.5±7.6	0.536
**DeMeester score (mean±SD)**	48±37	24±19	20±17	0.002
**Positive Symptom Index (%)**	25(52.1)	4(22.2)	2(9.5)	0.001
**Acid exposure time (% time with esophageal pH < 4) (mean±SD)**	7.3±2.9	4.3±2.7	3.9±2.8	0.001
**Symptom association probability (SAP) (%)**	32(66.7)	4(22.2)	3(16.7)	0.001
**Total reflux episodes (mean±SD)**	74±22	36±20	34±19	0.020
**Acid reflux episodes (mean±SD)**	45±18	24±21	20±16	0.015
**Weakly acidic reflux episodes (mean±SD)**	28±22	16±10	15±12	0.060
**Proximal reflux episodes (%)**	52.6±12.3	25.3±7.4	22.3±8.6	0.008

NERD, non-erosive reflux disease FH, functional heartburn AET: acid exposure time BMI: Mean body mass index SAP: Symptom association probability

### Evaluation of HRE findings

**Fig 2:**
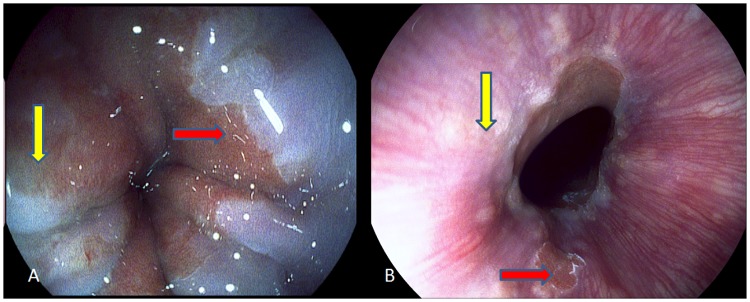
Minimal changes of NERD patients diagnosed using i-scan endoscopy with esophageal enhancement. A. Blurring Z-line (yellow arrow), uneven and friable mucosa at the squamo-columnar junction (red arrow). B. columnar islands (red arrow) and white mucosal turbidity (yellow arrow) was seen above the Z-line.

Patients with NERD showed more minimal change lesions in distal esophageal epithelium on HRE with i-scan imaging (Fig 2). The Z-line changes included blurring, irregular, tongue like extensions, islands and zigzag appearance (Fig 2A). Thirty-six out of 48 (75%) NERD patients showed one or more Z-line changes, which were more common than that of FH group (50%) and controls (47.6%; P = 0.040). Blurring or irregular Z-lines in 28 NERD patients (58.3%) were the most common i-scan endoscopic findings of minimal changes in this study.

Esophageal mucosa near gastro-esophageal junction had uneven and rough appearance with white mucosal turbidity, edema and friability (Fig 2B). As shown in Table 2, Thirty-five out of 48 (72.9%) NERD patients showed one or more minimal mucosal changes which were significantly higher than that of FH group 7/18 (38.9%) and controls 9/21 (42.8%; *p* = 0.014). White mucosal turbidity was the most common endoscopic findings (23/48, 47.9%) of minimal mucosal changes in NERD patients in this study.

**Table 2:** Frequency of minimal changes in NERD group, FH group and controls.

Minimal changes	NERD (n, %)	FH (n, %)	Control (n, %)	*p* value
**Change of Z-line shape**	36/48(75)	9/18(50)	10/21(47.6)	0.040
**Change of esophageal mucosa**	35/48(72.9)	7/18(38.9)	9/21(42.8)	0.014
**At least one positive minimal change**	42/48(87.5)	12/18(66.6)	13/21(61.9)	0.004

NERD, non-esophageal reflux disease. FH, functional heartburn

In total, there were 42/48 (87.5%) NERD patients with at least one or more minimal changes in this study, which significantly higher than that of FH patients (12/18, 66.6%) and controls (13/21, 61.9%; *p* = 0.004). These results showed that one minimal esophageal change or more had an accuracy of 80.3% [95% confidence interval (CI) 70–89 in diagnosing NERD patients from those with heartburn with 87.5% sensitivity (95% CI 82–94), 61.1% specificity (95% CI 56–72), 85.7% positive predictive value (95% CI 78–92) and 64.7% negative predictive value (95% CI 60–75; Table 2).

### Histopathological findings

Routine histological examination of the esophagus was performed in 42 NERD patients and 14 FH patients. Microscopic changes in the distal esophageal epithelium were observed. No eosinophilia or eosinophilic gastroenteritis were found in this study. Histological scores were used for evaluating microscopic changes between the two groups (Table 3). NERD patients had significantly higher average scores of intercellular spaces dilation (2.82±0.9 vs.1.2±0.6, *p* = 0.005) and papillae elongation (2.65±1.0 vs.1.5±0.8, *p* = 0.014), but not for basal cell proliferation (1.6±1.3 vs.1.0±0.9, *p* = 0.070). The histological score of the NERD patients was 7.1±1.2, which was higher than that of FH patients (3.4±1.0, *p* = 0.004; Table 3.

**Table 3:** Histopathological characteristics of patients with non-erosive reflux disease (NERD) and functional heartburn (FH) in relationship to diagnosis.

Microscopic changes	NERD (n = 48)	FH (n = 18)	*p* value
**Dilated intercellular spaces**	2.82±0.9	1.2±0.6	0.005
**Elongation of the papillae**	2.65±1.0	1.5±0.8	0.014
**Proliferation of basal cells**	1.6±1.3	1.0±0.9	0.070
**Histopathological sum score**	7.1±1.2	3.4±1.0	0.004

### Multivariate analysis

To better evaluate discrimination among the three subgroups, we added multivariate regression analysis for the pH/impedance features, HRE and histologic changes. The result showed that there were significant correlations of acid reflux with minimal changes of the *esophagus* (r = 0.752, *p*<0.001) and histological score (r = 0.724, *p*<0.001).

## Discussion

NERD and FH share common manifestations, which makes it difficult to distinguish between them without performing more invasive tests [30, 31]. In this retrospectively cohort study, we showed that the proportion of total reflux episodes and acid exposure time in NERD patients were higher than that of FH group and healthy controls. The results were similar to that observed by Savarino et al. [32]. The percentage of reflux episodes reaching the proximal esophagus in NERD patients was also greater than that of FH patients and controls.

Conventional assessment of impedance–pH monitoring includes AET and SAP/SI evaluations. However, because SAP and SI are determined by chance occurrences when reflux rates are low [33], clinical value of SAP and SI for distinguishing FH from GERD has been questioned in recent years. Recently, more sensitive makers have been explored by several gastroenterologists. Frazzoni et al. [34] reported that combined assessment of the post-reflux swallow-induced peristaltic wave index and the mean nocturnal baseline impedance allows objective diagnosis of hypersensitive esophagus independently of and significantly more often than that of using SAP and SI. Kandulski et al. [35] performed a prospective study by measuring intraluminal baseline impedance. They showed that measurement of baseline impedance in the lower esophagus can differentiate patients with erosive reflux diseases or NERD from patients with FH (78% sensitivity and 71% specificity), and therefore should be considered as a diagnostic tool for patients with proton pump inhibitor-refractory reflux. Hence, in clinical practice, when SAP and SI offer uncertain results, the post-reflux swallow-induced peristaltic wave index and levels of baseline impedance should be analyzed to avoid overlooking FH diagnoses.

In this study, Z-line minimal changes were seen in 75% NERD patients, whereas it was only seen in 50%of FH patients and 47.6%ofcontrols. When minimal changes were analyzed by using more than one endoscopic finding, same results were found between the two groups with sensitivity of 87.5% and positive predictive value of 85.7%. This was consistent with Rey JW et al.’s result [18] but not that of Netinatsunton et al.’s study [20]. Rey and his colleges performed high definition endoscopy with i-scan imaging and Lugol’s solution for the detection of inflammation in NERD patients. They showed 82.5%sensitivity and 100% positive predictive values for i-scan endoscopy in detecting minimal erosive reflux disease. The difference may be due to lack of standardized definition for minimal changes, lack of definitive gold standard test for minimal changes, and different study populations, endoscopic instruments and imaging technologies [10, 20, 36]. Therefore, multi-center prospective studies need to be conducted with standard definitions and more accurate diagnostic tools in the future.

Compromised esophageal mucosal integrity is now considered an etiology for GERD. Dilated intercellular space (DIS) of the esophageal epithelium is a sensitive marker for tissue damage in GERD patients and is the most appropriate marker for mucosal damage evaluation in NERD patients [37, 38]. Morphologic changes in esophageal mucosa observed using transmission electron microscopy, confocal laser microendoscopy and standard histopathologic evaluations have been reported in animal experiments and several clinical studies, which allows one to distinguish NERD from FH [29, 30, 39]. Kandulski et al. used histopathological sum scores to differentiate FH patients from NERD patients with high statistical significance (*p*< 0.0001). A cut-off value of ≥5 distinguishes NERD and FH patients with 85% sensitivity and 64% specificity [29]. In our study, using histological scores, we documented morphologic changes using light microscopy. Our results showed that NERD patients had much higher scores than did the FH group. This is consisting with our previous work of measuring intercellular spaces by using transmission electron microscopy [40].

To the best of our knowledge, there were no more published studies using this histologic score for the diagnosis of NERD and FH. Savarino et al. [30] evaluated histological scores in esophageal biopsies of NERD, FH patients and healthy controls by light microscopy. Based on the scores, they were able to differentiate NERD patients from FH patients with 79% accuracy 74% sensitivity and 86% specificity. They found no difference in the prevalence of microscopic esophagitis between FH patients and healthy controls. Although we did not have exact measurements of intercellular spaces in the current study, our result demonstrated pathologic changes correlated with acid reflux in NERD patients but not with FH and control subjects. Similar results had been reported in PPI-refractory NERD patients with heartburn when compared with FH patients [29, 35]. These findings suggest that esophageal biopsies using standard histopathologic evaluation are useful in differentiating NERD from FH. In addition, we performed multivariate regression analysis to analyze the relationships among pH/impedance features, HRE and histologic changes. The result showed that there were significant correlations between acid reflux with minimal changes of the esophagus (r = 0.752, *p*<0.001) and histological scores (r = 0.724, *p*<0.001).

The present study has several limitations. First, esophageal manometry studies were not performed in these subjects. According to the Rome III criteria, FH patients may have esophageal motility disorders. Savarino et al. [41] reported that esophageal motility disorders were present in 4% of FH and NERD patients, and their prevalence increases according to the GERD severity. However, esophageal manometry was not widely used in clinical practice during that time frame in China. Second, we did not analyze the characteristics of patients with hypersensitive esophagus. Another limitation was lack of data after PPI treatment in patients with positive i-scan endoscopy. It is unclear whether standard PPI treatment reverses abnormal findings on i-scan endoscopy. A previous study showed that 6-month omeprazole treatment completely restored DIS [42], the primary microscopic manifestation in NERD patients, which indicated that positive endoscopic findings are likely to diminish after PPI therapy. In this study we calculated the accuracy of endoscopy by using pH/impedance for diagnosing patients with heartburn, but the later methodology has limitations and needs further validation in larger studies in the future.

In conclusion, there is large overlap of NERD and FH in patients with heartburn but negative conventional endoscopy. I-scan endoscopy is a simple and useful tool for diagnosing minimal changes in NERD patients. Minimal changes could be useful markers to support clinical diagnosis of NERD in patients with heartburn. Combined 24-h MII-pH monitoring and i-scan endoscopy could distinguish NERD patients from those with FH.
